# An Effective Machine Learning for Prioritizing Hearing Loss Screening in Older Adults: A Primary Care Approach

**DOI:** 10.1111/jgs.70634

**Published:** 2026-07-31

**Authors:** Simone Seixas da Cruz, Edilson José Rodrigues, Michelle de Santana Xavier Ramos, Débora Conceição Santos de Oliveira, Paulo José dos Santos de Matos, Julita Maria Freitas Coelho, Dóris Firmino Rabelo, Alexandre Marcelo Hintz, Johelle Santana Passos‐Soares, Ana Claudia Morais Godoy Figueiredo, Isaac Suzart Gomes‐Filho

**Affiliations:** ^1^ Federal University of Recôncavo da Bahia (UFRB) Santo Antônio de Jesus Bahia Brazil; ^2^ Federal University of ABC São Bernardo Do Campo São Paulo Brazil; ^3^ State University of Feira de Santana (UEFS) Feira de Santana Bahia Brazil; ^4^ Federal Institute of Education, Science and Technology of Bahia (IFBA) Salvador Bahia Brazil; ^5^ Federal University of Bahia (UFBA) Salvador Bahia Brazil; ^6^ Federal District Health State Department Epidemiology Surveillance Brasília Brazil

**Keywords:** decision trees, older adults, predictive modeling, primary health care

## Abstract

**Background/Objectives:**

The high prevalence of hearing impairment in older adults and the scarcity of diagnostic resources in primary health care (PHC) highlight the need for solutions based on artificial intelligence. This study aimed to develop and evaluate a decision tree model to prioritize hearing examinations in this population.

**Methods:**

An observational study was conducted using 60 clinical profiles. The decision tree model was developed in R, using the Recursive Partitioning and Regression Trees (rpart) package after data preprocessing that included cleaning, dichotomization, and multiple imputations by chained equations (MICE). The algorithm was trained on 70% of the sample and tested on the remaining 30%. Its performance was evaluated using standard metrics.

**Results:**

The model achieved satisfactory performance with good accuracy, sensitivity, and specificity. The area under the curve (AUC) was 0.825, outperforming a classic logistic regression model (AUC 0.700). Based on the model's key predictors, which include the presence of tinnitus, not having a partner, female sex, hypertension and/or diabetes, and age ≥ 65 years, a user‐friendly, interactive web‐based dashboard was developed for clinical use.

**Conclusion:**

The decision tree model is a promising tool that demonstrates technical viability for prioritizing hearing loss screening. While further large‐scale external validation is required, it serves as a foundational step for integrating AI‐driven prioritization in primary health care.

## Summary

- Key Points
  ○ A pilot machine learning model based on decision trees was developed to identify older adults at higher risk for hearing loss.
  ○ The model achieved a high area under the curve (AUC) of 0.825, outperforming traditional logistic regression in predicting hearing impairment.
  ○ Key predictors for prioritization in primary care include tinnitus, lack of a partner, female sex, hypertension/diabetes, and age ≥ 65 years.
- Why does this paper matter?
  ○ This study presents a practical, interpretable, and effective tool that can be used in primary care to optimize the allocation of scarce resources for hearing screening, ultimately improving auditory care for older adults and supporting clinical decision‐making.

## 1 Introduction

The older adult population has shown significant growth worldwide, a phenomenon accompanied by an increase in chronic and degenerative diseases, including hearing acuity loss [1]. Hearing impairment in older adults is a public health problem that significantly affects quality of life, interfering with communication, socialization, and autonomy [2, 3].

Access to auditory diagnostic examinations, such as audiometry and immittance testing, is still limited in Brazil, especially in primary health care (PHC), due to the scarcity of available diagnostic resources [4, 5]. It is known that the development of optimized models that use artificial intelligence (AI) to analyze various factors and identify critical variables, aiming to predict clinical outcomes, represents an important approach to elevate the quality of general health services, including the auditory condition in older adults [6, 7, 8].

Initiatives aimed at developing innovative machine learning systems to assist in the prioritization of hearing examinations for older adults deserve recognition, as they can integrate high computing technology with the daily needs of public health, offering practical and effective tools for primary health care professionals.

Currently, hearing screening in PHC is conducted in a non‐standardized manner, varying according to the availability of local resources. In many municipalities, only anamnesis is used, which contributes to delayed referrals and an overload on specialized services. The protocol proposed in this work seeks to assist PHC professionals through a decision support system capable of generating a prioritization score based on clinical and sociodemographic variables, allowing for the identification of the most urgent cases for audiometric examinations. The scarcity of diagnostic resources in PHC thus highlights the need for innovative methods for prioritization, especially in areas with limited infrastructure [9].

The convergence between AI and the field of health has allowed deep learning algorithms to process large volumes of data, resulting in quick and accurate diagnoses [6, 10, 11, 12].

Despite significant ethical and regulatory challenges, AI‐based systems have considerable potential to transform future healthcare systems, especially in the field of auditory health, from optimizing workflow to prioritizing examinations, facilitating their implementation even in primary care settings [13]. It is known that such algorithms can identify anomalies and patterns with performance comparable to or even superior to that of specialists, contributing to more assertive diagnoses [14, 15, 16]. The implementation of various machine learning techniques, such as deep belief networks (DBN), logistic regression (LR), support vector machine (SVM), and Multilayer Perceptron (MLP), has shown good accuracy in identifying auditory problems [9].

By identifying a low‐cost and highly accurate method, especially in the field of auditory health, for more urgent cases requiring audiological examinations, it is possible not only to improve the quality of life of older adults by allowing them to receive a faster diagnosis and treatment but also to represent a significant step toward operational effectiveness in the care and management of the unified health system [7].

This study's main objective was to develop and evaluate a pilot decision tree model to demonstrate the proof‐of‐concept for prioritizing older adults who need a hearing examination in PHC, with the hypothesis that the clinical and sociodemographic characteristics of these individuals allow for an adequate prediction of the need for hearing examinations, thereby improving referrals in PHC. Thus, a model trained with these data will have the potential to classify priority individuals, with performance superior to conventional screening. Additionally, the secondary objectives included as follows: (a) Identifying the main factors associated with hearing loss; (b) comparing the performance of the decision tree model with a classic approach for dichotomous outcomes—LR; and (c) developing a prototype practical tool for the prioritization of audiometric examinations in older adults in PHC.

## 2 Method

### 2.1 Implementation of Machine Learning Algorithm(s)

This study developed a technological tool using machine learning to assist in better defining priority criteria for indicating hearing examinations among older adults in PHC, considering that diagnostic resources for hearing impairment are scarce and difficult to access. The tool was developed under the supervision of health/epidemiology/data science professionals, as well as with the participation of a speech‐language pathologist, all of whom are members of the research team.

### 2.2 Database

The database has been under development since March 2023, as described in the project “Loss of Auditory Acuity in Older Adults, Race and Ethnicity, and Other Associated Factors” (PVD53‐2021), registered at the Federal University of Recôncavo da Bahia. The database brings together sociodemographic, lifestyle, general health, and auditory condition variables, ensuring the confidentiality and anonymity of participants in compliance with ethical and regulatory guidelines.

### 2.3 Hearing Loss Assessment—Response Variable

Pure‐tone audiometry was defined as the gold standard for hearing loss assessment, and information related to this examination is part of the database. The cutoff point for severe hearing loss was considered when the older adult required a volume greater than 70 dB to hear a specific tone, according to the criteria established by Lloyd and Kaplan (1978) [17]. This objective definition allows for a standardized classification of hearing impairment and substantiates the prioritization of examinations, guiding referral strategies in PHC.

### 2.4 Data Preprocessing (Data Wrangling)

After data import, the preprocessing stage began with the objective of ensuring the integrity and consistency of the dataset for modeling. Initially, the data underwent a cleaning process that included the removal of duplicates and the exclusion of records with missing values in a key variable, ensuring that only complete information was considered.

Next, an exploratory analysis was performed to check the distribution of variables of interest, in order to guide subsequent transformations. To standardize the dataset, transformation operations were applied, such as the dichotomization of categorical variables and the conversion of labeled variables to factors, ensuring that the data were appropriately formatted for later analyses.

Considering the low proportion of missing data (< 1%), the multiple imputation by chained equations (MICE) technique was used, with the Predictive Mean Matching method in five iterations, preserving the original data distribution and avoiding the loss of relevant information. This approach, recognized in the biomedical literature for reducing the bias associated with case exclusion [18], was applied only to sociodemographic or clinical variables that were already anonymized, ensuring the ethical and scientific integrity of the study.

After the data manipulation and cleaning stage, the variables were dichotomized and defined to facilitate the interpretation of the results according to Table 1. For continuous variables, such as age, ROC curve analysis was performed to identify the most informative cutoff point, which was set at 65 years. This dichotomization allowed the data to be aligned with the requirements of the decision tree model, maintaining clinical interpretability and favoring the practical applicability of the results.

**TABLE 1:** Definition of variables of interest.

Variable name	Categories
Hearing loss^1^	Yes, No
Female Sex	Yes, No
Race/Ethnicity	Brown/Black, White
Age ≥ 65 years	Yes, No
With a partner	Yes, No
Has children	Yes, No
Hypertension	Yes, No
Diabetes	Yes, No
Presence of tinnitus	Yes, No
Lives alone	Yes, No
Schooling level	Up to 4 years, > 4 years

Abbreviation: dB, decibels.

^1^ Hearing loss > 70 dB–the volume of sound required for a person to hear a tone.

### 2.5 Machine Learning Model Construction

The algorithm of choice was initially the classic decision tree technique. This approach was selected due to its proven efficiency in classification problems, allowing for the segmentation of data into homogeneous groups based on predictor variables. Furthermore, decision trees offer easy interpretability of results, a fundamental aspect when dealing with applications in the health field, where a clear understanding of the factors influencing the decision is essential for clinical decision‐making.

For implementation, the model was developed in the R programming language, using the Recursive Partitioning and Regression Trees (rpart) package [19]. Initially, alternative predictive algorithms such as Random Forest (with ntree = 500) and SVM were explored. However, due to the small training set (*n* = 42), the bootstrap sampling and high‐dimensional mapping led to significant instability and lack of convergence. Consequently, a single pruned decision tree was prioritized to ensure stable results and clinical transparency. To mitigate the risk of overfitting and ensure model stability given the sample size (*N* = 60), the decision tree was developed using a Hold‐out validation approach (70% training and 30% independent testing). Strict hyperparameter constraints were applied as follows: A complexity parameter (cp) of 0.01, a minimum of 10 observations required to attempt a split (minsplit), and a maximum tree depth of 4. These measures were taken to prevent the model from capturing noise and to prioritize clinical interpretability.

This package is widely used in building decision trees due to its robustness and flexibility, allowing the algorithm to be adjusted according to desired control parameters (such as cp, minsplit, and maxdepth) to prevent overfitting and ensure result generalization. This algorithm adopted a recursive approach to divide the data into as homogeneous groups as possible based on hearing loss (the response variable). Divisions were made to maximize the purity of the child nodes relative to the parent node. Thus, the use of R and “rpart” enabled the creation of an interpretable and efficient model to classify the data for proper prioritization of hearing examinations.

### 2.6 Configuration Parameters

The model was configured using the argument method = “class,” which indicates that the problem is one of classification. This setting instructs the algorithm to treat the response variable as categorical, allowing for the construction of a tree that separates observations into distinct classes.

Furthermore, the cp (cp = 0.01) was defined. This parameter controls the penalty associated with each new split in the tree, helping to prevent the formation of excessively complex structures and, consequently, the occurrence of overfitting. In other words, a higher cp value limits the growth of the tree and favors a more generalizable solution.

A minimum number of observations for a split (minsplit = 10) was also stipulated. This means that a node could only be split if it contained at least 10 observations. This criterion ensures that the divisions made by the tree are based on a sufficient amount of data, contributing to the stability and reliability of the model.

To further control the model's complexity, the maximum depth (maxdepth = 4) of the tree was set. This limitation prevents the tree from growing indefinitely, restricting the number of levels and thus helping to avoid overfitting to the training data.

It is noteworthy that the impurity criterion adopted for splits in the decision tree was the Gini index, which is the default for the “rpart” function when using the classification method in the R software. This index quantifies the heterogeneity of the nodes, with lower values indicating greater homogeneity among observations [20]. With each split, the algorithm seeks to minimize the Gini index by selecting the cutoff points that maximize the reduction in impurity. In this way, the divisions lead to the formation of increasingly pure nodes, which is reflected in a more effective classification of hearing loss cases.

In other words, this approach enhances the interpretability of the tree, allowing for a clear identification of which factors contribute to group homogeneity, and ensures that the separations performed are the most informative for explaining the variability related to hearing loss, thereby contributing to the proper prioritization of specific examinations.

### 2.7 Model Training and Validation

The data were randomly divided into two sets as follows: 70% for model training and 30% reserved for testing. This division aims to ensure the reproducibility of the findings, allowing the model's performance to be consistently evaluated on data that were not used during its construction.

Furthermore, the k‐fold cross‐validation technique was applied during the training process, which allows for a more effective evaluation of the model's performance and helps prevent overfitting. This technique divides the training set into k subsets, and in each iteration, one of these subsets is used for validation while the rest are used for training, providing a reliable estimate of performance on unseen data.

To evaluate the model's effectiveness, various performance metrics were used, including accuracy, sensitivity, specificity, and predictive values. Additionally, the area under the curve (AUC) of the ROC curve was calculated, a metric that summarizes the model's overall discriminatory ability between groups with and without hearing loss, considering different classification thresholds.

### 2.8 Model Evaluation and Comparison

In addition to the decision tree, other approaches, such as LR, were preliminarily examined. However, the methodological detailing of the decision tree was prioritized due to its high interpretability and suitability for the purpose of this study. More in‐depth comparisons with other algorithms are reserved for future investigations, ideally with expanded databases.

### 2.9 Development of a Hearing Examination Prioritization Tool

Based on the findings of the best model, a technological tool was developed for the prioritization of hearing examinations in older adults in PHC. This tool allows health professionals to enter the sociodemographic and health characteristics of the older adults they serve through an interactive dashboard, generating a real‐time prioritization score that reflects the urgency for performing audiometric examinations. The values assigned to the predictor variables, derived from the final model, were carefully weighted to form a score that classifies the referral needs of service users.

Additionally, the application was submitted to a clinical evaluation with the help of a speech‐language pathologist, whose feedback allowed for adjustments and improvements in the tool's usability, thus ensuring its suitability for practical implementation in PHC.

### 2.10 Ethical Aspects

This study is linked to the project “Loss of Auditory Acuity in Older Adults, Race and Ethnicity, and Other Factors,” approved by the Research Ethics Committee [CEP] of the Federal University of Recôncavo da Bahia [UFRB] (Process No. 41064620.3.0000.0056—Annex 1), ensuring the confidentiality and anonymity of the participants. The data processing fully respected the principles of the General Data Protection Law (Law No. 13.709/2018), ensuring anonymization, exclusively scientific use, and restricted access to the research team.

## 3 Results

In this first application involving 60 complete clinical profiles, it was observed during preprocessing that less than 1% of the data was missing among the dichotomized variables sex, race/ethnicity, age, level of schooling, with a partner, having children, hypertension, diabetes, living alone, and presence of tinnitus. After multiple imputations using MICE (Predictive Mean Matching, 5 iterations), there was no statistically significant difference (McNemar's test, *p* > 0.05) in the proportions or variability of each category, which confirms the preservation of the original distribution (Table 2).

**TABLE 2:** Participant characteristics according to hearing loss status in older adults (*N* = 60).

Variables	Hearing loss	
	Yes *n* (%)	No *n* (%)
Female sex		
Yes	13 (27%)	28 (39%)
No	11 (23%)	8 (11%)
Race/Ethnicity		
Brown/Black	4 (8.3%)	9 (12.5%)
White	20 (41.7%)	27 (37.5%)
Age ≥ 65 years		
Yes	19 (39.6%)	21 (29%)
No	5 (10.4%)	15 (21%)
Not having a partner		
Yes	14 (29%)	13 (18%)
No	10 (21%)	23 (32%)
Has children		
Yes	23 (48%)	34 (47%)
No	1 (2%)	2 (3%)
Hypertension		
Yes	17 (35%)	24 (33%)
No	7 (15%)	12 (17%)
Diabetes		
Yes	9 (19%)	9 (12.5%)
No	15 (31%)	27 (37.5%)
Lives alone		
Yes	20 (42%)	25 (35%)
No	4 (8%)	11 (15%)
Presence of tinnitus		
Yes	18 (37.5%)	16 (22%)
No	6 (12.5%)	20 (28%)
Schooling level		
Up to 4 years	16 (39%)	25 (61%)
> 4 years	8 (42.1%)	11 (57.9%)

After randomly splitting the data into training (70%) and test (30%) sets, the configuration of the control parameters (cp = 0.01, minsplit = 10, and maxdepth = 4) proved effective in reducing overfitting, as evidenced by the consistency in the performance of the decision tree model. In the training set, the model achieved an accuracy of 73.81%, with a 95% confidence interval (95% CI): 57.96%–86.14%, a sensitivity of 80.77%, and a specificity of 62.50%. Furthermore, the positive and negative predictive values were 77.78% and 66.67%, respectively, and the balanced accuracy reached 71.63% (Table 3).

**TABLE 3:** Performance metrics of the decision tree model on training and test sets.

Metric	Training set (%)	Test set (%)
Accuracy	73.81	77.78
95% Confidence Interval	(57.96–86.14)	(56.10–93.59)
Kappa	40.92	43.04
Sensitivity	80.77	70.00
Specificity	62.50	87.50
Positive predictive value	77.78	70.00
Negative predictive value	66.67	87.50
Balanced accuracy	71.63	78.75

*Note:* Training set (*n* = 42); Test set (*n* = 18).

In the test set (Table 3), an accuracy of 77.78% was observed (95% CI: 56.10%–93.59%), with a sensitivity of 70.00% and a significant improvement in specificity, which reached 87.50%. The positive and negative predictive values were 70.00% and 87.50%, respectively, resulting in a balanced accuracy of 78.75%. These results suggest that the model has a good capacity to identify positive cases of hearing loss, although the reduction in sensitivity on the test set, compared to the training set, indicates a need for additional adjustments, possibly related to the current sample size.

Additionally, the ROC curve for the test set presented an AUC of 0.825, which indicates a good discriminative capacity for the model to identify cases of hearing loss. This value demonstrates that the algorithm can correctly distinguish between individuals with and without hearing loss in 82.5% of situations. These results reinforce the model's applicability as a screening tool in PHC. The matrices of confusion obtained from the training and test sets allowed for a detailed evaluation of the model's predictive performance (Tables S1 and S2). In the training set, consisting of 42 individuals, 26 did not have hearing loss: of these, 21 were correctly classified (true negatives) and 5 were incorrectly classified as having hearing loss (false positives). Among the 16 individuals with hearing loss, 10 were correctly identified (true positives) and 6 were misclassified, that is, without hearing loss (false negatives). In the test set, which had 18 individuals, 10 did not have hearing loss, of whom 7 were correctly classified (true negatives) and 3 were incorrectly classified, that is, with hearing loss (false positives). Among the 8 individuals with hearing loss, 7 were correctly identified (true positives) and 1 was misclassified, that is, without hearing loss (false negative). These results reinforce the model's ability to identify cases of hearing loss, although they still indicate the need for improvements to increase its predictive accuracy.

It is important to note that the confusion matrices presented in Tables S1 and S2 correspond to a specific training test partition, illustrating the model's hits and misses in that division. However, the metrics summarized in Table 3 obtained through cross‐validation reflected the average performance of the model across multiple data partitions. This approach lends greater robustness to the evaluation, which is why the values do not exactly match those observed in the confusion matrices.

To compare the performance of the decision tree model with another classic statistical model, a LR model was constructed with the same predictor variables. The performance of the models was compared based on the area under the ROC curve (AUC). The decision tree model had an AUC of 0.825, while LR obtained an AUC of 0.700 (Figure S1). The model's performance on the independent test set (AUC 0.825) was consistent with the training phase results. This stability suggests that the selected hyperparameters (cp and minsplit) effectively balanced model complexity and predictive power, even within a constrained sample. This result indicates that, in the current dataset, the decision tree model demonstrated a greater discriminative capacity between individuals with and without hearing loss. The performance difference suggests that the hierarchical and interpretable structure of the decision tree model may better capture the nonlinear relationships between the predictor variables and hearing loss, especially in contexts with a reduced number of observations and dichotomous variables. The decision tree model highlighted the variables presence of tinnitus, not having a partner, female sex, hypertension and/or diabetes, and age ≥ 65 years as the main predictors of hearing loss, as illustrated in Figure 1. These results provide a solid basis for future optimizations and for the practical application of the decision tree model in prioritizing hearing examinations in PHC. Attempts at comparison with other widely used predictive algorithms, such as Random Forest and SVM, were also made [21]. However, due to the reduced sample size, these methods presented convergence difficulties and instability in the results, which prevented an effective comparison with the decision tree model. Thus, it was only possible to perform a preliminary comparison with the LR model.

**FIGURE 1:**
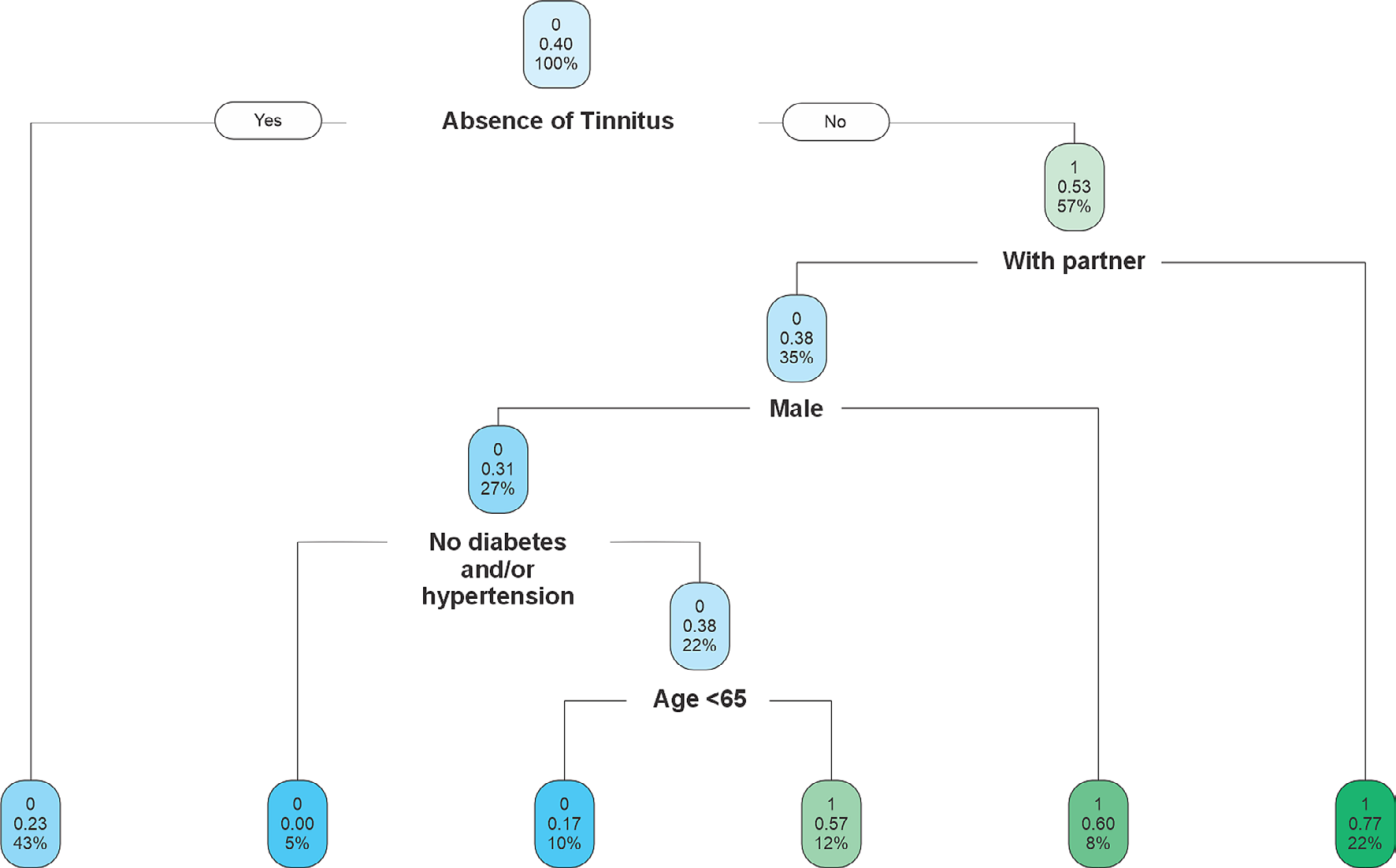
Pilot decision tree model for prioritizing hearing loss screening in older adults.

Despite the limitations of the decision tree model, a tool was constructed to facilitate the priority indication for hearing examination referral in PHC, the primary objective of the study. To this end, the variables were weighted according to their importance in the decision tree model (Table 4). The variables were ordered as follows: Presence of tinnitus, not having a partner, female sex, hypertension and/or diabetes, and age ≥ 65 years. The normalized weighting was calculated to ensure an adequate distribution of each variable's relevance, resulting in the following values: presence of tinnitus (3.33), not having a partner (2.67), female sex (2.00), hypertension and/or diabetes (1.33), and age ≥ 65 years (0.67). Based on this scale, which ranges from 0 to 10, the following cutoff points were suggested for the prioritization of hearing examinations: scores between 0 and 3.0 indicate low risk, scores from 3.1 to 7.0 indicate moderate risk, and scores above 7.0 indicate high risk. These intervals were established by considering an approximate division of the scoring spectrum into three categories, providing a clear and practical classification for health professionals, even though the cutoff points are preliminary and may be adjusted based on future empirical validations in PHC. Another objective of the present study was the development of a technological tool for the prioritization of hearing examinations in older adults. This work demonstrates the viability of the said tool for the prioritization of hearing examinations in older adults in PHC, implemented as an interactive webpage, using HTML, CSS, and JavaScript with Bootstrap support for a responsive and modern design, as well as html2pdf.js for automatic PDF report generation. The tool is hosted on the website https://sites.google.com/view/instrumentopesoasidosas/início. Through the interactive dashboard, health professionals can enter the clinical and sociodemographic data of the older adults and obtain, in real‐time, a prioritization score that indicates the urgency for referring them to hearing examinations.

**TABLE 4:** Weightings for prioritization of care variables in hearing loss diagnosis.

Variable	Initial value assignment (points)	Weighting^b^
Presence of tinnitus^a^	5	3.33
Not having a partner	4	2.67
Female sex	3	2.00
Hypertension and/or diabetes	2	1.33
Age ≥ 65 years	1	0.67

^a^ Variable considered most important in the model.

^b^ Weighting calculated for normalization to a total score of 10.

## 4 Discussion

This study successfully demonstrated that a machine learning model, specifically a decision tree, can effectively prioritize older adults in need of a hearing examination within the context of primary health care (PHC). The model achieved robust performance, with an AUC of 0.825, confirming its strong discriminative capacity. The results highlighted key clinical and sociodemographic variables as primary predictors of hearing loss, including the presence of tinnitus, not having a partner, female sex, hypertension, and/or diabetes, and age ≥ 65 years. These findings are consistent with previous studies in the auditory health literature, which have also identified these factors as significant risk indicators [22, 23, 24].

The methodological approach adopted in this study stands as a key strength. The choice of a decision tree model was strategic, prioritizing interpretability and transparency over the “black box” nature of more complex algorithms. In a clinical setting, where the trust and understanding of healthcare professionals are paramount, the ability to clearly explain the factors influencing a prioritization score is essential. This approach enhances the tool's credibility and facilitates its practical adoption. Furthermore, the rigor of our data handling and validation methods strengthens the study's reliability. The use of multiple imputations by chained equations (MICE) ensured that missing data were managed without introducing bias, a crucial step for maintaining data integrity. Similarly, the implementation of *hold‐out* cross‐validation during training provided a reliable estimate of the model's performance on unseen data, mitigating the risk of overfitting, a common issue in machine learning studies with smaller datasets.

Another notable strength is the translational nature of this research. Unlike many predictive models that remain at a theoretical level, this study culminated in the development of a tangible, interactive web‐based tool. This instrument provides a practical and immediate solution for PHC professionals, offering a data‐driven score to guide patient referrals and optimize the allocation of scarce diagnostic resources. The superior performance of our decision tree model compared to a classic LR model (AUC of 0.825 vs. 0.700) further underscores the value of this approach, suggesting that the hierarchical structure of the decision tree is more effective at capturing complex nonlinear relationships between risk factors and hearing loss in older adults.

Despite these promising results, it is important to acknowledge the limitations of this study. The primary constraint is the reduced sample size, which is a significant limitation that may affect the stability of the estimates. However, we addressed this by implementing rigorous pruning and growth constraints on the decision tree to favor generalization. Consequently, this study should be interpreted as a proof‐of‐concept and pilot investigation, demonstrating the feasibility of using simplified ML models to optimize screening workflows in resource‐limited Primary Care settings. A significant limitation of this study is the lack of external validation. The model was validated using only an internal hold‐out dataset from the same population. Therefore, while our results demonstrate strong internal consistency and potential, they should be viewed as preliminary. Future studies are needed to conduct temporal or geographic validation in different Primary Health Care cohorts to ensure the model's generalizability across diverse populations.

This limitation also prevented us from conducting a robust comparison with other widely used predictive algorithms, such as Random Forest, Gradient Boosting Machines, and SVM, which require larger datasets to achieve stable and reliable results. Additionally, while the clinical evaluation with a single specialist was a valuable step, the proposed prioritization score cutoffs (low, moderate, and high risk) are preliminary and require further empirical validation in a real‐world PHC setting with a larger cohort of professionals and patients.

In conclusion, our decision tree model serves as a proof‐of‐concept for prioritizing hearing loss screening in resource‐constrained settings. Although it shows satisfactory discriminative capacity, external validation in larger, independent samples is a mandatory next step before its widespread clinical implementation.

## Author Contributions

**Simone Seixas da Cruz:** conceptualization, methodology, writing – original draft, resources, supervision, data curation, formal analysis. **Edilson José Rodrigues:** data curation, formal analysis. **Michelle de Santana Xavier Ramos:** data curation, formal analysis. **Débora Conceição Santos de Oliveira:** software, validation. **Paulo José dos Santos de Matos:** investigation, data curation. **Julita Maria Freitas Coelho:** visualization, formal analysis. **Dóris Firmino Rabelo:** resources, supervision. **Alexandre Marcelo Hintz:** conceptualization, formal analysis. **Johelle Santana Passos‐Soares:** validation, writing, review and editing. **Ana Claudia Morais Godoy Figueiredo:** methodology, writing, review and editing. **Isaac Suzart Gomes‐Filho:** conceptualization, methodology, writing, original draft.

## Funding

This study was supported by the Coordination for the Improvement of Higher Education Personnel (CAPES) (no. 001), Brazil, and by the National Council for Scientific and Technological Development (CNPq), under Call No. 29/2024 (Decit/SECTICS/MS).

## Conflicts of Interest

The authors declare no conflicts of interest.

## Supporting information


**Figure S1:** Comparative analysis of discriminative performance: decision tree versus logistic regression models (proof‐of‐concept). *Note:* The ROC (receiver operating characteristic) curves illustrate the trade‐off between sensitivity (true positive rate) and specificity (1—false positive rate) for the pilot models. The decision tree (orange line) achieved an AUC of 0.825, while the logistic regression (red line) achieved 0.700. The dashed diagonal line represents a random classifier (AUC = 0.500). Abbreviations: AUC, area under the curve; ROC, receiver operating characteristic.
**Table S1:** Confusion matrix for the training set pilot model.
**Table S2:** Confusion matrix for the test set pilot model.

## Data Availability

The data that support the findings of this study are available on request from the corresponding author. The data are not publicly available due to privacy or ethical restrictions.
