# Supplementary material for: An Effective Machine Learning for Prioritizing Hearing Loss Screening in Older Adults: A Primary Care Approach
Source: J Am Geriatr Soc. 2026 Jul 31;74(10):2939–46. doi: 10.1111/jgs.70634 (PMC13723199; doi:10.1111/jgs.70634)
Supplement: Supplementary file 1 [file JGS-74-2939-s001.pdf]

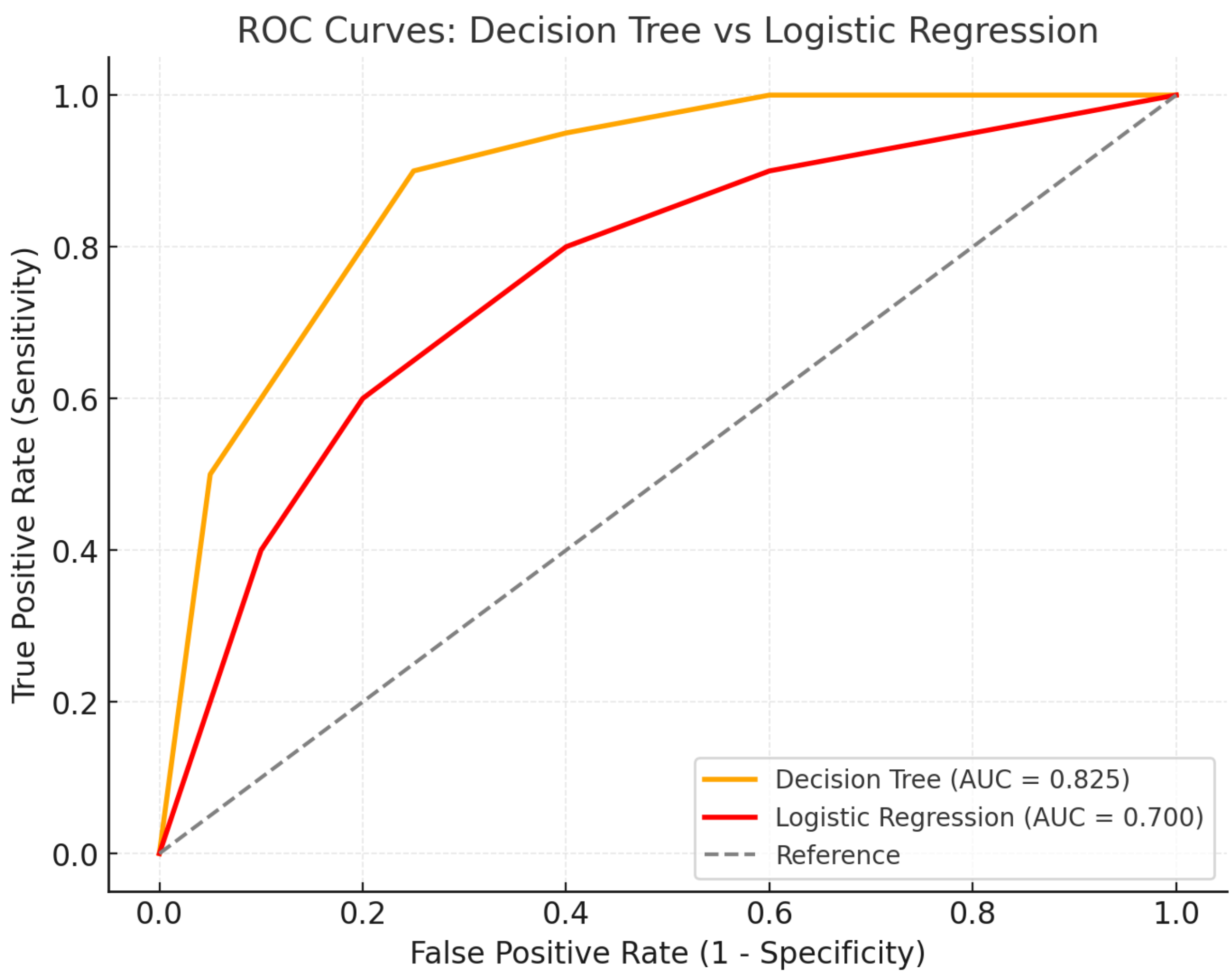

Supplementary Figure 1. Comparative Analysis of Discriminative Performance: Decision Tree vs. Logistic Regression Models (Proof-of-Concept).

Note: The ROC (Receiver Operating Characteristic) curves illustrate the trade-off between sensitivity (True Positive Rate) and specificity (1 – False Positive Rate) for the pilot models. The Decision Tree (orange line) achieved an AUC of 0.825, while the Logistic Regression (red line) achieved 0.700. The dashed diagonal line represents a random classifier (AUC = 0.500).

Abbreviations: AUC, area under the curve; ROC, receiver operating characteristic.

Supplementary Table 1. **Confusion Matrix for the Training Set Pilot Model.**

Gold Standard	Prediction: No Hearing Loss	Prediction: With Hearing Loss	Total
No Hearing Loss	21	5	26
With Hearing Loss	6	10	16

Note: The matrix shows the distribution of true positives (10), true negatives (21), false positives (5), and false negatives (6) used to calculate the performance metrics in Table 3. "Gold Standard" refers to the clinical audiometric diagnosis used as the reference for model evaluation.

Supplementary Table 2. Confusion Matrix for the Test Set Pilot Model.

Gold Standard	Prediction: No Hearing Loss	Prediction: With Hearing Loss	Total
No Hearing Loss	7	3	10
With Hearing Loss	1	7	8

Note: The matrix shows the distribution of true positives (7), true negatives (7), false positives (3), and false negatives (1) used to calculate the performance metrics in Table 3. "Gold Standard" refers to the clinical audiometric diagnosis used as the reference for model evaluation.
